# Effects of sphingolipids overload on red blood cell properties in Gaucher disease

**DOI:** 10.1111/jcmm.15534

**Published:** 2020-08-07

**Authors:** Lucie Dupuis, Caroline Chipeaux, Emmanuelle Bourdelier, Suella Martino, Nelly Reihani, Nadia Belmatoug, Thierry Billette de Villemeur, Bénédicte Hivert, Fathi Moussa, Caroline Le Van Kim, Marine de Person, Mélanie Franco

**Affiliations:** ^1^ UMR_S1134 BIGR, Inserm Institut National de Transfusion Sanguine Laboratoire d'Excellence GR‐Ex Université de Paris Paris France; ^2^ CNRS Institut de Chimie Physique UMR 8000 Université Paris‐Saclay Orsay France; ^3^ AP‐HP, CRML Maladies Lysosomales Service de Médecine Interne Hôpital Beaujon Université de Paris Clichy France; ^4^ APHP.6 CRML Maladies Lysosomales Service de Neuropédiatrie, Hôpital Trousseau Sorbonne Université Paris France; ^5^ Service d'Hématologie Hôpital Saint Vincent de Paul GHICL Lille France

**Keywords:** enzyme replacement therapy, erythropoiesis, Gaucher disease, red blood cells, sphingolipids

## Abstract

Gaucher disease (GD) is a genetic disease with mutations in the *GBA* gene that encodes glucocerebrosidase causing complications such as anaemia and bone disease. GD is characterized by accumulation of the sphingolipids (SL) glucosylceramide (GL1), glucosylsphingosine (Lyso‐GL1), sphingosine (Sph) and sphingosine‐1‐phosphate (S1P). These SL are increased in the plasma of GD patients and the associated complications have been attributed to the accumulation of lipids in macrophages. Our recent findings indicated that red blood cells (RBCs) and erythroid progenitors may play an important role in GD pathophysiology. RBCs abnormalities and dyserythropoiesis have been observed in GD patients. Moreover, we showed higher SL levels in the plasma and in RBCs from untreated GD patients compared with controls. In this study, we quantified SL in 16 untreated GD patients and 15 patients treated with enzyme replacement therapy. Our results showed that the treatment significantly decreases SL levels in the plasma and RBCs. The increased SL content in RBCs correlates with abnormal RBC properties and with markers of disease activity. Because RBCs lack glucocerebrosidase activity, we investigated how lipid overload could occur in these cells. Our results suggested that SL overload in RBCs occurs both during erythropoiesis and during its circulation in the plasma.

## 1 INTRODUCTION

Gaucher disease (GD) is a lysosomal storage disease that is caused by a mutation in the enzyme glucocerebrosidase (GCase), which is implicated in sphingolipid catabolism. GBA deficiency in GD is characterized by the accumulation of the GBA substrates glucosylceramide (GL1) and glucosylsphingosine (Lyso‐GL1) and secondary metabolites such as sphingosine (Sph) and sphingosine‐1‐phosphate (S1P). GD is subdivided into three clinical subtypes: GD type 1 is the chronic non‐neuronopathic form that is characterized by hepatomegaly, splenomegaly, anaemia, thrombocytopenia, and bone manifestations as bone infarcts, avascular necrosis, fractures, lytic lesions and osteoporosis^1^; and types 2 and 3 represent the acute and chronic neuronopathic forms, respectively. Treatments such as enzyme replacement therapy (ERT) with recombinant GCase or substrate reduction therapy (SRT) by reducing glycosphingolipid substrates are available for GD type 1 patients.

Clinical manifestations in GD patients have been associated with monocyte/macrophage dysfunction because accumulation of GCase substrates is observed in these cells that transformed into Gaucher cells, and they are thought to be responsible for the major GD symptoms. However, GD pathophysiology is complicated and not fully understood. Several studies suggested that other cell types may contribute to GD pathophysiology.^2^, ^3^, ^4^, ^5^, ^6^, ^7^

Previously, we showed that red blood cell (RBC) membrane abnormalities may contribute to the occurrence of complications in GD, such as ischemic events and anaemia.^3^ This was supported by the abnormal rheological, morphological and membrane adhesion properties that were observed in RBCs from GD type 1 patients.^3^, ^8^ We also showed that ERT allowed the recovery of these RBC properties.^8^ In addition, we demonstrated that erythropoiesis is affected in Gaucher patients,^9^ and iron metabolism is affected in patients with hyperferritinemia.^10^ In summary, our data highlighted the role of erythroid cells as contributors to GD pathophysiology.

To decipher the underlying mechanisms leading to RBC abnormalities, we investigated the lipid composition in GD RBCs and erythroid progenitors. It has been previously shown that glucosylceramide (GL1) accumulates in GD RBCs^11^ and GL1 and glucosylsphingosine (Lyso‐GL1) accumulates in the plasma in GD.^12^, ^13^, ^14^ We also recently reported higher levels of several sphingolipids GL1, lyso‐GL1, sphingosine (Sph), and sphingosine‐1‐phosphate (S1P) in the plasma and in RBCs from untreated (UT) GD patients compared with healthy controls.^15^ We have been suggested that abnormal lipid composition in RBCs from GD patients are mainly responsible for the abnormal RBC properties contributing GD pathophysiology. To further elucidate the role of RBC in GD, we investigated whether ERT normalizes the sphingolipid (SL) content in plasma and RBCs. We then studied the effects of velaglucerase alfa on the SL content in plasma and RBCs from 31 GD patients who were either treated or not treated with ERT (comparative study) and from seven GD patients before and after ERT (pre‐post study).

Here, we report that ERT decreases the SL level in both plasma and RBC compartments. Significant correlations between SL overload and abnormal RBC properties as well as markers of disease activity, strongly suggest that SLs represent critical mediators of RBC defects in GD. Because mature RBCs lack GCase activity, we investigated whether lipid overload in RBCs occurs by passive diffusion between the plasma compartment and the circulating RBCs and/or during the erythropoiesis process.

## 2 MATERIALS AND METHODS

### 2.1 Patients

GD patients were recruited prospectively between February 2012 and April 2015. Three centres participated in the study (Beaujon Hospital and Trousseau Hospital, Assistance Publique‐Hôpitaux de Paris, and Saint Vincent Hospital, Lille, France). Blood samples were collected in EDTA‐containing tubes from 31 GD patients (30 GD type 1 patients; one GD type 3 patient which was revealed during this study; and 11 healthy volunteers who were over 18 years old). None of the GD patients were splenectomized. The untreated group (GD UT) was composed of 16 patients who had never received ERT when blood samples were collected. Nine of these patients did not fit the criteria for treatment according to the guidelines of the French GD committee. The other seven patients were treated using ERT at the time of blood sampling just before the first infusion, and during the few months after the study.

The ERT‐treated (GD ERT) group included 15 patients who were treated with ERT for at least 1 year. All treated patients received velaglucerase alfa (VPRIV, Shire Human Genetic Therapies) intravenously every 2 weeks. The GD UT and GD ERT groups were analysed and compared in the comparative study. Seven of 16 GD UT patients were followed for at least 1 year after starting the velaglucerase alfa treatment, and blood samples were collected. Pre‐ERT and post‐ERT groups were analysed and compared in the longitudinal study. Detailed demographic, clinical and biological data from the comparative and the longitudinal studies are provided in Tables 1 and 2, respectively. Clinical and biological data were provided by the haematology laboratory of the corresponding hospital.

**Table 1:** Comparative study: demographic characteristics, haematological and biological features of untreated and ERT‐treated GD patients

	GD UT	GD ERT	*P* value
n	16	15	
Male	8	9	
Age (y)	19.5 [3‐60]	32 [5‐80]	
Mutations			
N370S heterozygous	13	12	
N370S homozygous	1	1	
D409H homozygous	1	1	
Undetermined	1	1	
Organomegaly			
Hepatomegaly	10 (63%)	2 (13%)	.009
Splenomegaly	14 (88%)	2 (13%)	<.0001
Bone involvement			
Disease history	7 (44%)	8 (53%)	.72
At sampling date	3 (19%)	5 (33%)	.43
Biological measurements			
Hb (g/dL)	12.45 [10.8‐14]	14.8 [12.3‐17.3]	<.0001
Htc (%)	35.35 [30.4‐41.4]	42.5 [35‐51]	<.0001
Platelet count (10^3^/mm^3^)	100.5 [57‐208]	157 [69‐373]	.009
CCL18 (pg/mmol)	663 [149‐991]	185 [85‐518]	.01
Chitotriosidase activity (nmol/h/mL)	8058.5 [517‐17 502]	582 [173‐9400]	.002
Red blood cells properties			
RBCs deformability EI at 1.69 Pa	0.225 [0.196‐0.25]	0.272 [0.203‐0.295]	.0012
RBCs abnormal morphology (%)	2.845 [1.02‐5.49]	0.59 [0.28‐4.4]	<.0001

Note

The characteristics and biological data for 16 untreated GD patients who had never received ERT (GD UT) were compared with data from 15 patients treated with ERT for at least one year. Bone involvement was considered to be significant for patients who experienced at least one bone crisis, bone pains or radiologic (MRI) skeletal manifestations including bone infarcts, avascular osteonecrosis, fractures and lytic lesions in their disease history or as new bone event at the time of sampling. The values are expressed as the median [extremes] and (percentages). The *P* values were determined using the Mann‐Whitney test to compare continuous variables between GD UT and GD ERT patients. Fisher's exact test was used to analyse categorical data in samples, that is splenomegaly and hepatomegaly. All characteristic and biological parameters, that is splenomegaly, hepatomegaly, Hb, Htc, CCL18 (C‐C motif chemokine ligand 18) and chitotriosidase activity were usually obtained on the day of blood sampling or within the previous few weeks. Analyses of RBCs properties, that is elongation index (EI) at 1.69 Pa as well as pourcentage of RBCs exhibiting abnormal morphologies were performed on fresh blood at the day of sampling.

Abbreviations: Hb, haemoglobin; Htc, hematocrit.

**Table 2:** Longitudinal study: clinical and biological data

n	7
Male	3
Age (y)	23 [3‐52]
Mutations	
N370S heterozygous	6
N370S homozygous	0
D409H homozygous	1
R163X/I206M	0
Undetermined	0

	Pre‐ERT	Post‐ERT	*P* value
Organomegaly			
Hepatomegaly	7 (100%)	1 (14%)	.0047
Splenomegaly	7 (100%)	0	.0006
Bone involvement			
At sampling date	2 (33%)	2 (33%)	ns
Biological measurements			
Hb (g/dL)	12.9 [11.1‐13.8]	14.4 [12.3‐15.3]	.03
Htc (%)	36.3 [30.4‐39.3]	40.9 [35‐43.4]	.03
Platelet count (10^3^/mm^3^)	91 [57‐163]	191 [106‐262]	.02
CCL18 (pg/µmol)	722 [663‐765]	150 [85‐317]	ns (.12)
Chitotrisidase activity (nmol/h/mL)	13 964 [1470‐17 500]	435 [62‐8765]	.02

Note

The characteristics and biological data for 7 GD patients were compared before (pre‐) and at least one year after (post‐) ERT (pre‐post ERT) with velaglucerase alfa. We considered bone involvement as significant for patients with bone pains and/or radiologic (MRI) skeletal manifestations including bone infarcts, avascular osteonecrosis, fractures and lytic lesions as new bone event at the time of sampling. The values are expressed as the median [extremes] and (percentages). The *P* values were determined using the Mann‐Whitney test to compare continuous variables between GD UT and GD ERT patients. Fisher's exact test was used to analyze categorical data in samples, that is splenomegaly and hepatomegaly. All characteristic and biological parameters, that is splenomegaly, hepatomegaly, Hb, Htc, platelet count, CCL18 (C‐C motif chemokine ligand 18) levels and chitotriosidase activity were usually obtained on the day of blood sampling or within the previous few weeks.

For one patient followed up during the first 120 days of ERT, we collected blood samples just before the first infusion and at 26, 40 54, 79 and 121 days after beginning treatment.

The study was conducted in accordance with the Declaration of Helsinki and was approved by the local ethics committee. Patients provided informed consent.

### 2.2 Analysis of RBC properties

All the experiments on RBC morphology and deformability were performed on fresh blood sample, as previously described.^3^ Giemsa‐stained blood smears were performed to quantify RBCs exhibiting abnormal morphologies. RBC deformability was determined at 37°C by laser diffraction analysis (ektacytometry) using the laser‐assisted optical rotational cell analyzer (LORRCA, Mechatronics).

### 2.3 Chemical and reagents for lipid quantification

Methanol, formic acid and tert‐butyl methyl ether (MTBE) were purchased from Carlo Erba. Ammonium formate was purchased from Sigma‐Aldrich. d‐Glucosyl‐β1‐1′‐N‐tetracosanoyl‐d‐erythro‐sphingosine (GL1; d18:1/24:0) from Matreya LLC. d‐Glucosyl‐β1‐1′‐d‐erythro‐sphingosine (Lyso‐GL1; d18:1), d‐erythro‐sphingosine (Sph; d18:1), d‐erythro‐sphingosine 1‐phosphate (S1P; d18:1), and the internal standards (IS) C17‐d‐erythro‐sphingosine (C17B; d17:1), C17‐d‐erythro‐sphingosine‐1‐phosphate (C17‐S1P; d17:1) and N‐heptadecanoyl‐d‐erythro‐sphingosine (C17Cer; d18:1/17:0) were obtained from Avanti Polar Lipids.

### 2.4 Sample preparation and UHPLC‐MS/MS analysis for sphingolipid quantification

After blood collection, the plasma and RBCs were frozen at −80 and −196°C, respectively, as previously described.^3^, ^16^ RBC samples were thawed and resuspended in ID‐CellStab (Bio‐Rad) 24 hours before the lipid extraction, and plasma samples were thawed just before lipid extraction. Protein precipitation and total lipid extraction were performed according to our previously described method.^15^

A reverse‐phase UHPLC‐MS/MS method was developed and validated for the rapid and simultaneous determination of the four SLs in both plasma and RBCs.^15^ UHPLC‐MS/MS experiments were performed on an Ultimate 3000 HPLC system coupled to a TSQ Quantum Ultra mass spectrometer that was equipped with an ESI ion source (Thermo Scientific). The SL levels in the plasma and RBCs from GD patients were determined as previously described.^15^ Briefly, separations were performed on a 50 × 2.1 mm, 1.8 µm Acquity UPLC HSS T3 column, which was protected with a 5 × 2.1 mm, 1.8 µm pre‐column that was filled with the same stationary phase. Eluent A consisted of a pH 2.5, 1 mmol/L formate buffer, and eluent B was pure methanol containing the same concentration of formate buffer. The targeted SLs were eluted with the following gradient: from 80% B to 100% B over 2 minutes, then the mobile phase composition was held constant for 6 minutes. All experiments were performed at 30°C at a flow rate of 0.5 mL/min. Quantification was set to the selecting reaction monitoring (SRM) mode. Data acquisition and processing were performed using the Xcalibur data system.

Sphingolipids quantification was determined in plasma and RBCs (with n = 6 replicates). We converted the units of concentration to nmol per litre of whole blood. For this, we took into account the hematocrit (Htc) level of each patient. The concentrations found in the RBC were multiplied by the Htc and those determined in the plasma were multiplied by [1 − Htc].

### 2.5 Exogenous sphingolipid addition to control blood samples

Blood samples from a healthy volunteer were freshly collected and experiments were performed in glass haemolysis tubes. The four SLs (GL1, Lyso‐GL1, S1P and Sph) were simultaneously added to the blood sample and incubated for a maximum of 2 hours at 37°C. At t = 0 minute (t0), the 4 SLs were added to the blood at a known concentration, and the sample was incubated for 15 minutes (t15). The plasma and the RBCs were then separated by centrifugation at 4°C. This step was repeated after 30 minutes (t30) and after 2 hours (t2 hours) of incubation. The samples at t0, t15, t30 and t2 hours were then frozen for subsequent determination of the SL concentrations in the plasma and in the RBCs. The haemoglobin A assay with the Drabkin reagent (Sigma‐Aldrich) was performed to standardize the SL quantification in each thawed RBC sample.

The concentration of added SL was determined to mimic the SL composition in the plasma of GD patients. For this, we took into account the levels that were previously found by Chipeaux et al^15^ in 2017 for the median values of SL that were measured in the controls and the maximum values that were measured in samples from GD patients in this study. We subtracted the maximum value of the SL that was measured in the plasma of the GD group from the median value of the same SL that was measured in the plasma of the control group (CTL group). The optimal concentrations to be added to reach the maximum concentration that was measured in the plasma of the Gaucher patient were provided in Table S2.

### 2.6 Effect of the GCase inhibitor CBE during in vitro erythropoiesis

CD34^+^ cells were isolated from the peripheral blood of a healthy donor using a Pancoll density gradient followed by positive selection using anti‐CD34 antibodies (Miltenyi CD34 Progenitor Cell Isolation Kit) and midi‐MACS columns. CD34^+^ cells were subjected to an expansion phase (4 days) in IMDM medium (Gibco cell culture) with Stem Cell Factor (SCF, 10 ng/mL), heparin (25 ng/mL), interleukin (IL)‐3 (10 ng/mL), BIT 9500 (15%), AB human serum (3%) and human plasma (2%), followed by a differentiation phase (18 days) in IMDM medium with SCF (1 ng/mL), heparin (10 ng/mL), IL‐3 (1 ng/mL), Erythropoietin (Epo, 3 μg/mL), BIT 9500 (15%), AB human serum (3%) and human plasma (2%). On day 7, IL‐3 was removed from the culture medium, and Conduritol B Epoxyde (CBE; Santa Cruz) at 1 mmol/L or H_2_O was added and renewed every 3 days. On days 7, 10, 14 and 18, differentiation steps were followed by flow cytometry using APC‐conjugated Alpha4 (CD49d, BD Bioscience), FITC‐conjugated Band3 (BRIC6, IBGRL Research Products) and Pe‐Cy7‐conjugated GPA (CD235a, BD Bioscience) antibodies. Before staining, cells were incubated in FBS supplemented in 2% BSA for 30 minutes at 4°C to block non‐specific antibody binding sites by the Fc receptors, and then, they were incubated with antibodies in PBS supplemented in 0.2% BSA for 30 minutes at 4°C. Acquisition was performed with a BD FACS Canto II flow cytometer (BD Bioscience), and analysis was performed using flowJo software.

Differentiation was also followed by analysing the cell morphologies by microscopy (Nikon Eclipse Ti‐S microscope). For this, a suspension of 10^5^ cells was collected for cytospin preparation then stained with May‐Grünwald‐Giemsa (MGG) solution (MGG analysis). Cells were counted using the 60× A/L oil objective, and the number of proerythroblasts, basophilic, polychromatic, acidophilic and reticulocytes was expressed as the percentage of total cells. On days 7, 14 and 18, 20 × 10^6^ cells were collected and resuspended in 1 mL of a sucrose solution (250 mmol/L) with Tris (5 mmol/L) and EDTA (1 mmol/L) at pH 7.5 for total lipid extraction.

To test the efficacy of CBE, GCase activity was measured by flow cytometry using the fluorogenic GCase substrate PFB‐FDGlu (5‐(pentafluorobenzoylamino) fluorescein di‐β‐d‐glucopyranoside) (Life Technologies) after 48 hours of treatment. Cells were incubated for 15 minutes with 1 mmol/L of the substrate or DMSO as the control at 37°C and 5% CO_2_. The reaction was then stopped, and the percentage of positive cells was measured.

### 2.7 Statistical analysis

Non‐parametric tests were performed. For the comparative study, parameters were compared between two groups using a Mann‐Whitney *U* test. The Wilcoxon signed‐rank test was used to compare paired observations (longitudinal study). Spearman's correlation coefficient was used to determine the association between the SL quantification, RBC properties, and the biological parameters. Fisher's exact test was used to analyse categorical data in small samples. All results are given as two‐tailed *P* values, with *P* < .05 considered to be statistically significant.

## 3 RESULTS

### 3.1 Characterization of patients on ERT

All demographic descriptions as well as clinical and biological parameters for the comparative and the pre‐post ERT studies are presented in Tables 1 and 2, respectively. As expected, the response to ERT in organomegaly included large decreases in the size of the spleen and the liver. ERT treatment significantly increased the haemoglobin (Hb) and Htc values and the platelet count (Table 1). ERT‐treated patients (GD ERT) exhibited significantly lower chitotriosidase activity and a lower level of the CCL18 chemokine compared with untreated patients (GD UT). Similar results were observed in the pre‐post study except for the CCL18 level (Table 2). For bone involvement, 81% of GD UT and 87% of GD ERT patients exhibited bone infiltration during the history of the disease (data not shown). Moreover, 44% of GD UT and 53% GD ERT patients experienced at least one bone crisis in their disease history. On the day of blood sampling or within the previous few weeks, eight GD patients experienced bone manifestations in the comparative study (Table 1), as well as in two patients from the pre‐post study (Table 2). However, the total number of GD patients exhibiting bone defects at the time of blood sampling was too small to draw a conclusion regarding the effect of ERT on bone disease.

### 3.2 Effect of ERT on sphingolipid levels in GD patients

Quantification of the SL levels in RBC and plasma from GD patients was performed using an UHPLC‐MS/MS method for simultaneous measurement of the SLs GL1, Lyso‐GL1, Sph and S1P. We previously showed, using this method, that these four SLs exhibited higher concentrations in the plasma and RBCs in untreated Gaucher patients compared with control individuals.^15^

**Figure 1:**
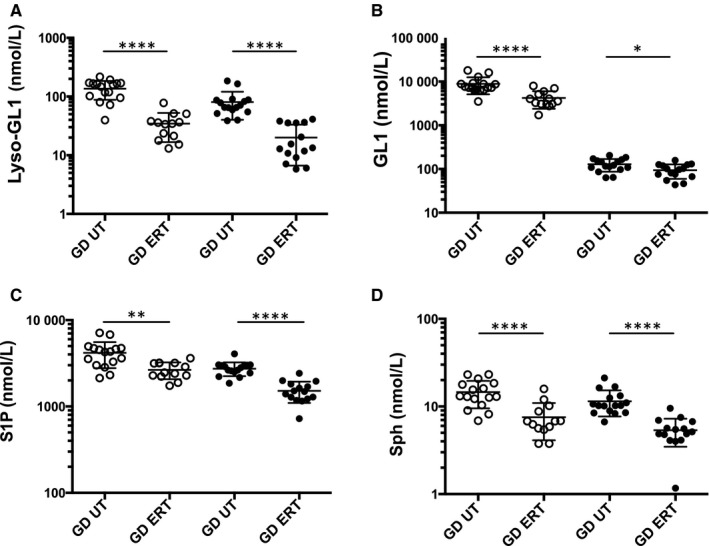
Enzyme replacement therapy (ERT) normalizes sphingolipid content in plasma and RBC in the comparative study. A, Lyso‐GL1, (B) GL1, (C) S1P and (D) Sph levels in plasma (open circles) and RBCs (dark circles) of GD untreated (GD UT, n = 16) and GD ERT‐treated (GD ERT, n = 15) patients. *P < .05, **P < .01, ****P < .0001

**Figure 2:**
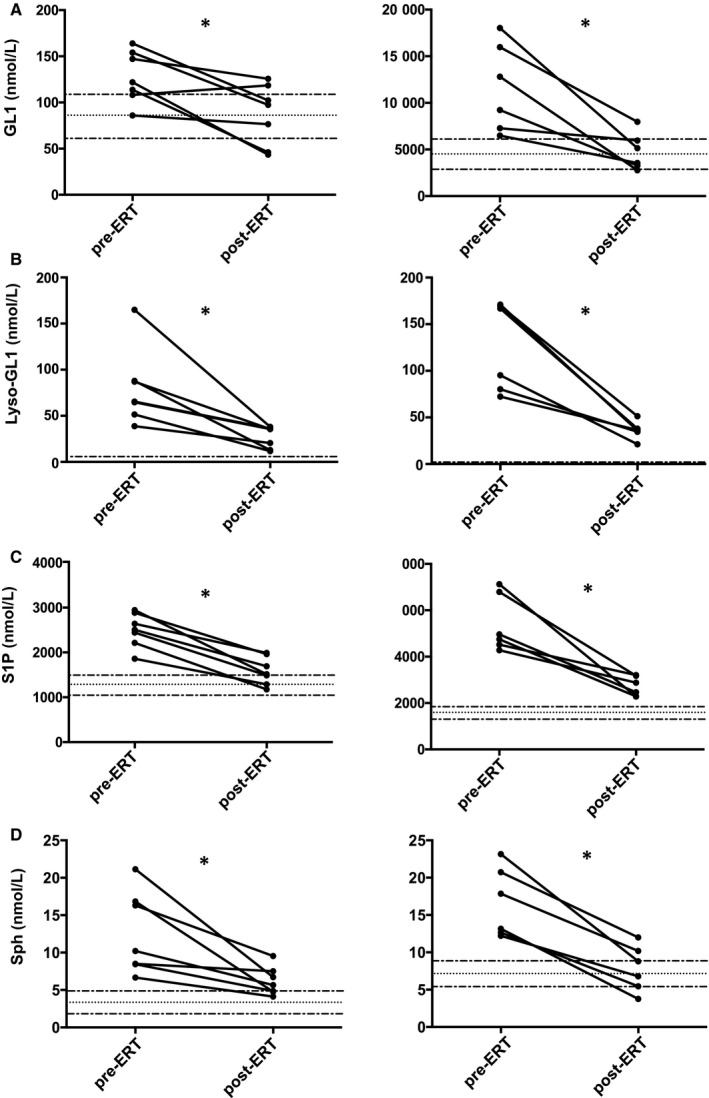
ERT reduces sphingolipid content in plasma and RBC in the longitudinal study. A‐D, The graphs on the left panel represent the SL levels (GL1, Lyso‐GL1, Sph, and S1P) in RBCs of GD patients pre‐ and post‐enzyme replacement therapy (ERT) (n = 7). *P < .05. The graphs on the right panel represent the SL levels (GL1, S1P, Lyso‐GL1 and Sph) in plasma of GD patients pre‐ and post‐enzyme replacement therapy (ERT) (n = 7). *P < .05. Dotted lines represent the median values and min/max values that were measured in control RBCs

In the comparative study, we showed that ERT treatment significantly decreased the plasma and the RBC levels of the four SLs (Figure 1). Similar results were observed in the longitudinal study for which the patients were followed before ERT and for at least 1 year after starting treatment (n = 7) (Figure 2 A–D left panels and right panels for SL measurements in the RBCs and in the plasma, respectively). However, after 1 year of treatment, ERT did not allow a return to normal values for some SL studied, and this was particularly the case for the SL levels measured in the plasma. Then, the results differed from one individual to another from a fully effective treatment after 1 year to an insufficient reduction in SL (Figure 2).

**Figure 3:**
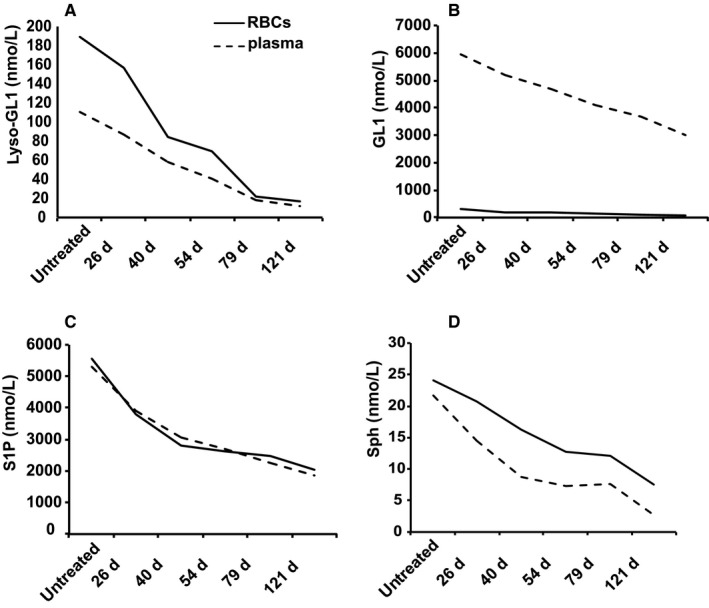
Follow‐up of a patient during the first 120 days of ERT. A decrease of the SL levels (GL1, Lyso‐GL1, Sph and S1P) in RBCs (full line) and plasma (dashed line) from a GD patient during the first 121 days of ERT

We had the opportunity to follow an untreated GD patient during the first 120 days of ERT. We measured SL levels in the plasma and RBCs on day 0 (before the first infusion of the recombinant enzyme) and on days 26, 40, 54, 79 and 121 after starting the treatment. This monitoring allowed us to observe a progressive and concomitant SL ‘discharge’ in both plasma and red cell compartments (Figure 3). As previously shown, the GL1 concentration in RBCs is lower compared with the GL1 plasma concentration. These results indicate that ERT decreases SL levels in both plasma and RBC compartments.

### 3.3 Correlation of sphingolipid levels in GD RBC with markers of disease activity

We investigated correlations between SL levels that were measured in the plasma and in the RBCs, and the markers of the disease activity as well as RBC properties.

As shown in Table S1, the increased concentrations of Lyso‐GL1, Sph and S1P in RBC significantly correlated with all the markers of the disease burden such as the level of plasma Lyso‐GL1 and CCL18, Hb levels and the chitotriosidase activity. As previously shown, GL1 content found in RBCs did not correlate with markers of GD such as the level of Hb,^15^ the level of plasma lyso‐GL1 and CCL18, and the chitotriosidase activity. However, the increased concentration of the four SLs measured in the plasma correlated significantly with the decrease in Hb and with increased CCL18 and chitotriosidase activity (data not shown). GL1, Lyso‐GL1, Sph and S1P levels in RBCs or in the plasma did not correlate with the platelet count (Table S1 and data not shown, respectively). This difference in the platelet count had already been observed in previous studies.^14^

We also investigated the correlation between SL levels that were measured in the RBCs and their membrane properties. As previously described,^3^ abnormal RBC morphology and deformability that were observed in the untreated GD patients were normalized by ERT in the comparative study (Table 1) and in the pre‐post study (data not shown). Significant correlations were found between the SL overload in the two compartments and the percentage of RBC exhibiting abnormal morphology or decreased deformability, except for the GL1 levels in the RBC (Table S1 and data not shown). We also observed that patients with the highest degree of RBC abnormal properties exhibit important SL accumulation in plasma and in RBCs (data not shown). From all these data, we concluded that SL overload in GD RBC correlated with markers of GD activity and with the defects observed in RBC from GD patients.

### 3.4 Effect of exogenous sphingolipid overload on control RBCs

We previously investigated the amounts of SL in the plasma and mature RBC from GD patients compared with healthy volunteers. We measured similar concentrations of Lyso‐GL1, Sph, and S1P in both compartments, suggesting that these SLs can be freely incorporated into the RBC by passive diffusion.^15^ The inability of GL1 to be incorporated into RBCs is most probably because of its steric hindrance.^15^

To test this hypothesis, we performed incubations of normal RBCs with plasma‐containing high concentrations of the four SLs that are equivalent to those measured in Gaucher patients (Table S2). Then, we performed kinetic of measurements for each SL in the plasma and RBCs after up to 2 hours of incubation.

**Figure 4:**
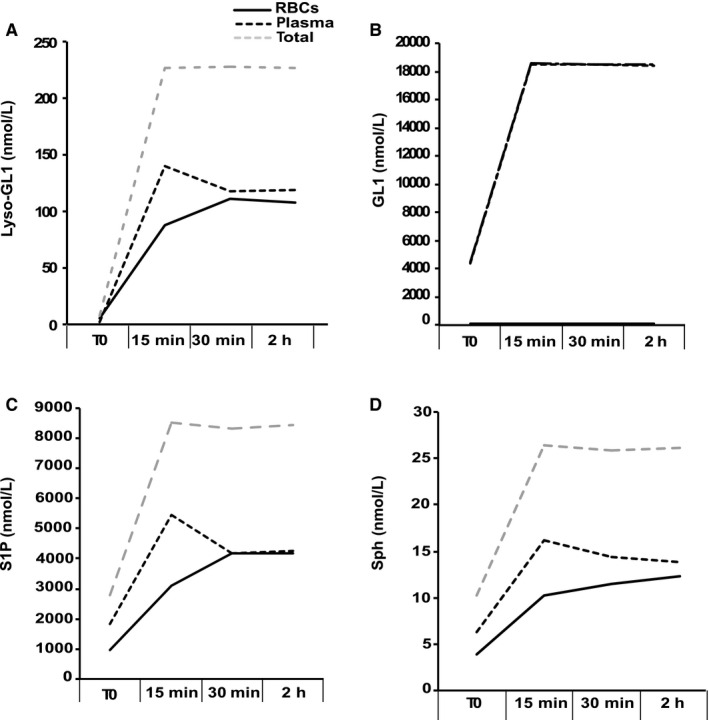
Effect of exogenous sphingolipids added to control blood samples on SL content in RBCs. Graphs show representative results (n = 2) of the kinetic measurement of the four SLs (Lyso‐GL1, GL1, S1P and Sph in A, B, C and D, respectively) before (T0) and 15, 30 min, and 2 h after they were added on control blood samples in the RBC compartment (full lines), in the plasma compartment (dark dashed lines), and in both compartments (grey dashed lines)

Our results, shown in Figure 4, confirmed that the SLs Lyso‐GL1, Sph and S1P could be gradually incorporated by RBCs until reaching the equilibrium concentrations after 2 hours of incubation. However, exogenous GL1 was not incorporated into RBCs. The SL levels measured in the RBC compartment at the end of the experiment did not reach the maximum value measured in Gaucher patients (Table S2). However, all these results support the hypothesis of diffusion from the plasma to the RBC, at least for 3 SL.

### 3.5 Sphingolipid quantification during erythropoiesis in CBE‐treated erythroblasts

Although passive diffusion from plasma may account for the abnormal presence of SL in RBCs, this mechanism cannot fully explain all the overloading. The GCase activity was detected in erythroblast progenitors, but not in circulating mature RBCs.^3^ This suggests that for GCase deficiency, as observed in GD, lipid accumulation could also occur during the early erythropoiesis stage. We then performed an in vitro erythropoiesis experiment in the presence or absence of CBE, a known specific GCase inhibitor, to mimic the GD context when the enzyme is still expressed in the cells. Erythroid differentiation was monitored from the pro‐erythroblast stage to the reticulocyte stage for 18 days. At day 7 of differentiation, CBE (1 mmol/L) was added and renewed every 3 days.

**Figure 5:**
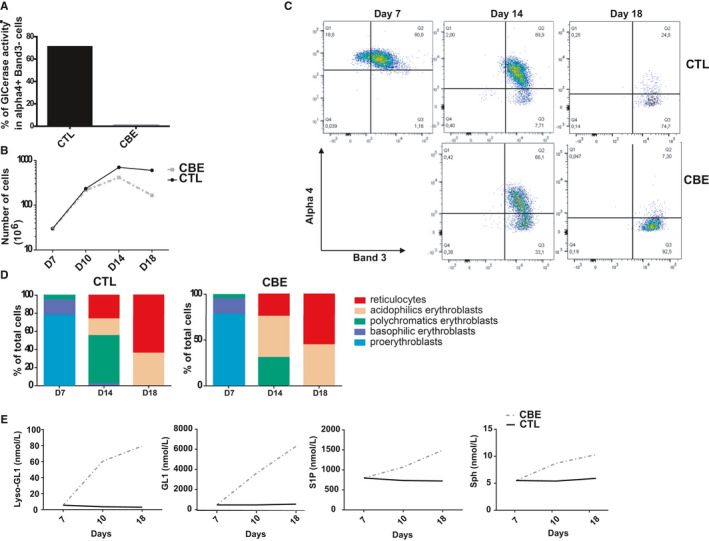
Effect of CBE during the in vitro erythropoiesis on the SL content. This Figure shows representative data (n = 2). A, The graph represents the percentage of cells with GCase activity in Alpha4^+^Band3^−^ erythroid progenitor subsets in control (CTL) and CBE‐treated (CBE) cultures. B, Erythroid cell proliferation: the graph represents the number of cells in control culture (CTL, dark full line) and CBE culture (CBE, grey dashed line) from day 7 to day 18 of differentiation. C, Flow cytometry analysis of alpha4 and Band3 cell surface expression at day 7, 14 and 18 of differentiation. Alpha4^+^Band3^−^ cells represent the early stage of differentiation and alpha4^−^Band3^+^ cells represent more mature stage of differentiation. D, Morphological analysis after May‐Grünwald‐Giemsa (MGG) staining. Graphs represent the percentage of each erythroid progenitor subset at days 7, 14 and 18 of differentiation for the control and CBE‐treated cultures. E, SL levels (GL1, Lyso‐GL1, Sph and S1P) measured over time in the control cell culture (CTL, dark full line) and in the CBE‐treated culture (CBE, grey dashed line). Lipids were measured using the UHPLC‐MS/MS method at days 7, 10 and 18 of differentiation

We first observed that the GCase activity was mainly detected (for about 80% of the cells) in the early stages of differentiation, corresponding to integrin alpha4^+^Band3^−^ erythroid progenitors from healthy controls (data not shown). We then measured the GCase activity in the absence or presence of the inhibitor at day 7 of differentiation. At this stage, we did not detect any GCase activity in the CBE‐treated cell culture compared with the non‐treated control culture (Figure 5A).

We then investigated the effect of the CBE inhibitor during erythropoiesis by analysing the proliferation and differentiation of the erythroid progenitors with or without CBE on days 7, 14 and 18 of culture. Our results showed a significant decrease in the proliferation of the CBE‐treated cells compared with control cells on day 14 and 18 of erythroid differentiation (Figure 5B). We also observed an increase in the percentage of differentiated cells, as evidenced by the increase in the percentage of the alpha4^−^Band3^+^ cell subpopulation at days 14 and 18 of differentiation. At day 14, 33% of the erythroid progenitors were alpha4^−^Band3^+^ in the CBE‐treated condition compared with 7.7% in the control condition (Figure 5C). On day 18, we observed 92.5% of alpha4^−^Band3^+^ cells in the CBE‐treated condition compared with 74.7% in the control condition (Figure 5C). Supporting these results, the morphological analysis of erythroid progenitors by MGG at day 14, showed a lower percentage of basophilic erythroblasts and a higher percentage of acidophilic and reticulocyte cells in the CBE‐treated condition compared with the control condition (Figure 5D).

All these results highly suggested a reduced cell proliferation and an accelerated erythroid differentiation when the GCase is inhibited. This is in agreement with our previous studies showing an abnormal erythroid differentiation in GD that occurred independently of the macrophage defects.^9^

We then measured the SL concentrations in the cell cultures on days 7, 10 and 18 of erythroid differentiation. As shown in Figure 5E, we first observed in the control condition that the four SL concentrations remained stable during erythroid differentiation. However, by day 10 of erythroid differentiation, an accumulation of the four SLs was observed when the erythroid progenitors were treated with CBE. At the end of the erythroid differentiation, we measured twice as many rates of Sph and S1P and a 14‐fold increase in the amount of Lyso‐GL1 and GL1 in the CBE‐treated cells compared with the control cells. This latter result indicated that the accumulation of the four SLs also occurred during erythropoiesis, at least in an in vitro GD‐mimicking condition.

## 4 DISCUSSION

Key SLs are known to accumulate in GD and are responsible for numerous aspects of their pathophysiology.^6^, ^12^, ^13^, ^14^, ^17^ The accumulation of plasma LysoGL1 of Gaucher patients has recently been described as the most relevant biomarker in GD.^14^, ^18^ However, the role of SL in the GD pathophysiology is not fully understood. Our previous findings revealed that RBC membrane abnormalities may contribute to the occurrence of complications in GD, such as ischemic events and anaemia.^3^ We have also recently demonstrated an unexpected macrophage‐independent ineffective erythropoiesis that could contribute to central anaemia in GD.^9^ These data strongly suggested that the erythroid compartment could be a key player in the pathophysiology of GD. We have been suggested that lipid overload in RBC from GD patients might be responsible for their abnormal properties.

Our recent studies showed an accumulation of the four SLs (Lyso‐GL1, GL1, Sph and S1P) in RBCs from GD patients compared with healthy individuals.^15^ We observed about twice as much GL1, Sph, and S1P and 14‐times more Lyso‐GL1 in Gaucher RBCs compared with control RBCs.^15^

These data suggested that abnormal RBC lipid composition in GD might be responsible for Gaucher symptoms involving erythroid cells. Supporting this hypothesis, our results showed that the ERT treatment by velaglucerase decreased the plasma and RBC levels of the four SLs. Moreover, the increased SL content in RBCs is highly correlated with abnormal RBC morphology and membrane deformability, strongly suggesting that these SLs represent critical mediators of RBCs abnormalities. This SL overload in RBCs also correlated significantly with the known markers of disease activity such as plasma Lyso‐GL1,^14^, ^18^ CCL18 levels,^19^ chitotriosidase activity^20^ and anaemia indicating that the lipid overload in RBCs is related to the disease activity. It could be interesting to investigate if there is a correlation between the lipid overload in RBCs and the spleen volume in untreated GD patients. In addition, although it is known that splenectomy increase the half‐life of circulating RBCs, it will be interesting to investigate the impact of the lipid overload in the erythrocyte in some splenectomized patients. Overall, future study of the potential contribution of RBCs to spleen dysfunction needs further investigations. Additionally, it would be interesting to perform the lipid profile of other sphingolipidosis where anaemia and splenomegaly is not a major clinical problem, but where SL also accumulate such as in Krabbe disease and Fabry disease.

The direct consequence of SL overload in GD on the RBC properties such as their deformability and their propensity to adhere to the endothelium is unknown and requires further investigation. However, the effect of several SLs on RBC morphology in other diseases was recently shown. One study demonstrated that patients with hypertension exhibited accumulation of phospholipids in their RBCs, leading to increased membrane fluidity and fragility.^21^ In addition, the altered SL metabolism in RBCs of mice that were deficient in hepatocyte nuclear factor (HNF1A) has been linked to changes in calcium homeostasis leading to an abnormal morphology of the RBCs, their fragility, and the consequent anaemia.^22^ It has also been demonstrated that S1P secretion by the specific transporter Mfsd2b is critical for RBC morphology.^23^

We then investigated the mechanisms leading to the accumulation of SL in the RBC membrane. Because mature RBCs lack GCase activity, we investigated whether lipid overload in mature RBCs could occur by passive diffusion between the plasma compartment and the RBCs and/or during the erythropoiesis process. Our data showed that the passive incorporation of Lyso‐GL1, Sph and S1P into RBCs could contribute to the SL overload in the RBCs of Gaucher patients. We also performed in vitro erythropoiesis experiments with CBE treatment to inhibit the GCase activity at the early stage of the erythroid differentiation. This experiment, which was performed in vitro in the presence of CBE, is in agreement with our previous results showing a dyserythropoiesis in the progenitors from GD patients, which was characterized by accelerated erythroid differentiation and decreased cell proliferation.^9^ Inhibiting the GCase during the erythroid differentiation led to accumulation of the four SLs in RBCs. Collectively, our data indicated that SL overload could take place during the erythropoiesis process and also throughout the RBC lifespan during their circulation in the plasma. RBCs are known to be the major source of plasma S1P, and previous studies demonstrated that this bioactive lipid is a positive regulator of erythropoiesis.^24^ The impact of SL overload on the erythropoiesis course needs to be further investigated in GD.

Taken together, our data strongly suggested that the SLs that accumulated during erythropoiesis and diffusion of Lyso‐GL1, Sph and S1P in erythrocytes could contribute to the SL overload that was measured in the RBCs of GD patients.

Sphingolipids are signalling molecules that are involved in cellular processes such as inflammation, differentiation, proliferation and apoptosis.^25^, ^26^ Their levels in the plasma and in RBCs are critical for regulating erythropoiesis and membrane integrity of RBCs. Because SL accumulation in the blood could directly contribute to RBC fragility, anaemia, and the inflammation observed in GD, the consequence of SL overload deserves also to be studied in a physiological context using a murine model.

Using the UHPLC‐MS/MS methodology, we showed, for the first time, the accumulation of four SLs in RBCs of GD patients. This new technology could represent a powerful tool to study lipid overload and signalling in RBC diseases and/or sphingolipidosis.

## CONFLICT OF INTEREST

The authors confirm that there are no conflicts of interest.

## AUTHOR CONTRIBUTION

**Lucie Dupuis:** Formal analysis (equal); Investigation (equal). **Caroline Chipeaux:** Formal analysis (equal); Investigation (equal). **Emmanuelle Bourdelier:** Investigation (equal). **Suella Martino:** Formal analysis (supporting); Investigation (supporting); Methodology (supporting). **Nelly Reihani:** Formal analysis (supporting); Investigation (supporting). **Nadia Belmatoug:** Formal analysis (supporting); Methodology (supporting). **Thierry Billette de Villemeur:** Formal analysis (supporting); Methodology (supporting). **Bénédicte Hivert:** Investigation (supporting). **Fathi Moussa:** Methodology (equal); Supervision (equal). **Caroline Le Van Kim:** Methodology (equal); Supervision (supporting); Writing‐original draft (supporting); Writing‐review & editing (supporting). **Marine de Person:** Investigation (equal); Methodology (equal); Supervision (equal); Writing‐original draft (equal). **Mélanie Franco:** Conceptualization (lead); Formal analysis (equal); Funding acquisition (lead); Investigation (equal); Methodology (equal); Writing‐original draft (lead); Writing‐review & editing (lead).

## Supporting information

Table S1

Table S2

## Data Availability

The data that support the findings of this study are available from the corresponding author upon reasonable request.
