# Supplementary material for: Effects of sphingolipids overload on red blood cell properties in Gaucher disease
Source: J Cell Mol Med. 2020 Aug 7;24(17):9726–36. doi: 10.1111/jcmm.15534 (PMC7520281; doi:10.1111/jcmm.15534)
Supplement: Supplementary file 1 — Table S1 [file JCMM-24-9726-s001.pdf]

Table 3. Correlation of SL dosage in RBC with biological and RBC parameters on Gaucher cohort.

	GL1	Lyso-GL1	Sph	S1P
Biological parameters				
**plasma lyso-GL1** *(nM)*	*0.16*^{ns}	*0.77*****	*0.61*****	*0.76*****
**Hb** *(g/dL)*	*-0.03*^{ns}	*-0.76*****	*-0.69*****	*-0.62*****
**CCL18** *(pg/µmol)*	*0.37*^{ns}	*0.52***	*0.49**	*0.47**
**Chitotriosidase** *(nmol/h/mL)*	*0.44*^{ns}	*0.62***	*0.65***	*0.53**
**platelet count** *(10^3/mm^3)*	*-0.40**	*-0.41**	*-0.41**	*-0.46***
RBC properties				
**abnormal morphology** *(%)*	*0.43**	*0.51***	*0.60*****	*0.46**
**deformability at 1.69Pa**	*-0.20*^{ns}	*-0.47**	*-0.55***	*-0.55***

Pearson coefficient (ρ) between the different parameters are shown as well as the corresponding p values (p <0.05, ** p <0.01, *** p <0.001 and **** p <0.0001).*
