# Supplementary material for: Effects of sphingolipids overload on red blood cell properties in Gaucher disease
Source: J Cell Mol Med. 2020 Aug 7;24(17):9726–36. doi: 10.1111/jcmm.15534 (PMC7520281; doi:10.1111/jcmm.15534)
Supplement: Supplementary file 2 — Table S2 [file JCMM-24-9726-s002.pdf]

Table 4. Summary of the concentrations measured in GD patients and controls as well as in our experience of adding exogenous lipids.

	Sphingolipid quantification in plasma (nM)				Sphingolipid quantification in RBCs (nM)			
	GL1	Lyso-GL1	S1P	Sph	GL1	Lyso-GL1	S1P	Sph
Summary of the results :								
Median in controls *(obtained from Chipeaux et al., 2017)*	**3844**	**1.6**	**1 609**	**6.9**	**82**	**5.2**	**1 274**	**4.0**
Maximum values measured in GD UT patient	**18021**	**217**	**7127**	**23**	**204**	**184**	**4071**	**21**
Exogenous lipids added on control blood :								
Concentration to add to control samples	**14000**	**220**	**5500**	**16**				
Concentration in RBCs at T0					**74**	**5.4**	**960**	**3.9**
Concentration in RBCs (2 hours of incubation)					**78**	**107**	**4168**	**12**
